# Mice deficient in protein tyrosine phosphatase receptor type Z (PTPRZ) show reduced responsivity to methamphetamine despite an enhanced response to novelty

**DOI:** 10.1371/journal.pone.0221205

**Published:** 2019-08-20

**Authors:** Akihiro Fujikawa, Yukihiro Noda, Hideko Yamamoto, Naomi Tanga, Gaku Sakaguchi, Satoko Hattori, Wen-Jie Song, Ichiro Sora, Toshitaka Nabeshima, Goro Katsuura, Masaharu Noda

**Affiliations:** 1 Division of Molecular Neurobiology, National Institute for Basic Biology, Higashiyama, Myodaiji-cho, Okazaki, Japan; 2 Division of Clinical Sciences and Neuropsychopharmacology, Faculty of Pharmacy, Meijo University, Shiogamaguchi, Tempaku-ku, Nagoya, Aichi, Japan; 3 Department of Psychiatry and Behavioral Sciences, Tokyo Metropolitan Institute of Medical Science, Kamikitazawa, Setagaya-ku, Tokyo, Japan; 4 School of Life Sciences, The Graduate University for Advanced Studies (SOKENDAI), Higashiyama, Myodaiji-cho, Okazaki, Aichi, Japan; 5 Biomarker R&D Dept., SHIONOGI & CO. LTD., Futaba-cho, Toyonaka, Osaka, Japan; 6 Division of Systems Medical Science, Institute for Comprehensive Medical Science, Fujita Health University, Dengakugakubo, Kutsukake-cho, Toyoake, Aichi, Japan; 7 Department of Sensory and Cognitive Physiology, Graduate School of Medical Sciences, Kumamoto University, Honjo, Chuo-ku, Kumamoto, Japan; 8 Department of Psychiatry Kobe University Graduate School of Medicine, Kusunoki-cho, Chuo-ku, Kobe, Hyogo, Japan; 9 Advanced Diagnostic System Research Laboratory, Fujita Health University Graduate School of Health Sciences, Dengakugakubo, Kutsukake-cho, Toyoake, Aichi, Japan; 10 Department of Psychosomatic Internal Medicine, Kagoshima University Graduate School of Medical and Dental Sciences & University Hospital, Sakuragaoka, Kagoshima, Japan; 11 Cell Biology Center, Institute of Innovative Research, Tokyo Institute of Technology, Nagatsuta-cho, Midori-ku, Yokohama, Kanagawa, Japan; University Paris Diderot, FRANCE

## Abstract

Methamphetamine (METH), a commonly abused drug, elevates extracellular dopamine (DA) levels by inducing DA efflux through the DA transporter (DAT). Emerging evidence in rodent models suggests that locomotor responses to a novel inescapable open field may predict behavioral responses to abused drugs; METH produces more potent stimulant effects in high responders to novelty than in low responders. We herein found that mice deficient in protein tyrosine phosphatase receptor type Z (*Ptprz*-KO) exhibited an enhanced behavioral response to novelty; however, METH-induced hyperlocomotion was significantly lower in *Ptprz*-KO than in wild-type mice when METH was administered at a non-toxic dose of 1 mg per kg body weight (bdw). Single-cell RT-PCR revealed that the majority of midbrain DA neurons expressed PTPRZ. No histological alterations were observed in the mesolimbic or nigrostriatal dopaminergic pathways in *Ptprz*-KO brains; however, a significant decrease was noted in brain DA turnover, suggesting functional alterations. *In vivo* microdialysis experiments revealed that METH-evoked DA release in the nucleus accumbens was significantly lower in *Ptprz*-KO mice than in wild-type mice. Consistent with this result, *Ptprz*-KO mice showed significantly fewer cell surface DAT as well as weaker DA uptake activity in striatal synaptosomes prepared 1 hr after the administration of METH than wild-type mice, while no significant differences were observed in the two groups treated with saline. These results indicate that the high response phenotype of *Ptprz*-KO mice to novelty may not be simply attributed to hyper-dopaminergic activity, and that deficits in PTPRZ reduce the effects of METH by reducing DAT activity.

## Introduction

Methamphetamine (METH) and amphetamine (AMPH) are sympathomimetic phenethylamine derivatives that exert central nervous system (CNS) stimulant effects by inducing, in part, the reversal of dopamine (DA) flow to the synaptic cleft through the DA transporter (DAT) [1–3]. Converging evidence from animal and human studies indicates that high novelty seekers are at a higher risk of using drugs of abuse than low novelty seekers [4, 5]; exposure to novelty activates the same neural substrate that mediates the rewarding effects of drugs of abuse, which may explain the close relationship between novelty seeking and drug seeking behaviors.

Experimental animals are categorized as either a “high responder” or “low responder” based on their levels of locomotor activity in a novel environment in an inescapable open field. Previous studies indicated that an acute injection of various drugs of abuse, including METH, AMPH, and cocaine (a DAT reuptake blocker), produces more potent locomotor stimulant effects in high responders than in low responders [5]. Combinational studies using local lesions induced by 6-hydroxydopamine (6-OHDA) and microdialysis to monitor extracellular DA levels revealed that the rewarding effects of these drugs were mostly dependent on the mesolimbic DA system of the brain [5, 6]. Therefore, individual differences in responses to novelty and abused drugs may be related to individual differences in mesolimbic DA functions. However, the common regulatory mechanism underlying novelty and drug responsiveness currently remains unclear.

Protein tyrosine phosphorylation is reversibly regulated by protein tyrosine kinases (PTKs) and protein tyrosine phosphatases (PTPs), and is involved in the regulation of various neuronal functions, such as neurotransmission [7], neurite growth, synapse formation [8], and transporter trafficking, including DAT [9, 10]. PTPRZ, one of the receptor-like protein tyrosine phosphatases (RPTPs), is predominantly expressed in the brain [11–13]. Our preliminary study on a *Ptprz*-knockout (KO) mouse cohort that was backcrossed for four generations (93.7% homogeneity with the C57BL6J genetic background) revealed altered responsiveness to novelty and METH, and we presented these results at the annual meeting of the Society of Neuroscience[14]: The knockout line was originally generated by gene targeting in 129X1/SvJ ES cells [15]. However, some studies subsequently demonstrated the importance of mouse strains for investigating psychostimulant-induced locomotor activity; for example, C57BL/6J mice show greater AMPH-induced locomotor activation and DA efflux in the striatum than 129S2/SvHsd mice [16].

In the present study, we therefore performed the study again using the fully backcrossed *Ptprz*-KO mouse cohort for ten or more generations to yield 99.9% genetic homogeneity, and verified the findings that *Ptprz*-KO mice exhibited an increased locomotor response to novelty, whereas their locomotor response to METH was reduced. Furthermore, we found that *Ptprz* deficiency may reduce cell surface DAT proteins, thereby reducing METH-evoked DA release.

## Materials and methods

### Ethics statement and experimental animals

All procedures in the present study were approved by the Institutional Animal Care and Use Committee of the National Institutes of Natural Sciences, Japan (approval numbers: 12A078, 13A172, 14A150, 15A095, 16A147, and 17A023) and performed in accordance with the guidelines of the Institutional Committee for the Use of Animals for Research. *Ptprz*-KO mice [15] were backcrossed with the inbred C57BL/6J strain (CLEA Japan) for more than ten generations. Mice were housed under specific pathogen-free (SPF) conditions at a constant room temperature (23°C) and 50–55% humidity with a 8:00 to 20:00 light cycle. Four to 5 weeks after birth, three to four sex-matched mice were housed in plastic cages (cage size: 12 × 21 × 12.5 cm) with paper-chip bedding, and food and water were provided *ad libitum*. Adult male mice (4 to 6 months old) were used in the present study. Mice were handled gently to minimize stress. Surgeries for implanting a guide cannula for brain microdialysis were performed under isoflurane anesthesia, 2% lidocaine cream was applied to the incision site after surgery for acute pain relief, and all efforts were made to minimize suffering. Behavioral experiments were performed during the light period (between 9:00 and 17:00) by at least two different individuals under blind conditions.

### Open field and novel object exploration tests

Tests were performed as described previously [17] with slight modifications as follows. Mice were placed in an open field (internal diameter, 75 cm and height, 40 cm) divided by a grid into twenty-five equal segments, and their locomotion (the number of crossings of the lines marked on the floor) was manually counted for 5 min per day on 2 consecutive days. The novel object test was started in the open field after habituation to the environment by repeated exposure. Mice remained in the open field without objects for 9 min, a white plastic cube (45 × 45 × 45 mm) was then placed in the center area, and their exploratory behavior (the number of crossings of the center) was recorded for a further 9 min.

### Locomotor activity measurements

Locomotor activity was measured in a clear acryl chamber (40×26.5×40 cm) with an activity monitoring apparatus (SCANET SV-40, Melquest).

### Drug treatments

METH (Methamphetamine hydrochloride, Dainippon Pharmaceutical) was dissolved at 0.1 mg/ml (for 1 mg per kg bdw injection) or 0.3 mg/ml (for 3 mg per kg bdw injection) with sterilized 0.9% NaCl (Normal saline, Otsuka Pharmaceutical). Apomorphine (cat no. A4393, Sigma) was dissolved at 0.1 or 0.3 mg/ml in 0.9% saline with 0.1% ascorbic acid (cat no. 012–04802, Wako Pure Chemical Industries) before use. Drugs were injected subcutaneously into the backs of mice.

### Quantification of DA and its metabolites in brain tissues

Mice were sacrificed by decapitation to avoid any complications due to anesthesia. Brain tissues were separated quickly as described previously [18], placed on an ice-cooled glass plate, weighed, and homogenized with 10 mM HClO_4_, 0.1 mM sodium pyrosulfite, and 20 μM ethylenediaminetetraacetic acid (EDTA) disodium salt containing 1 ng/ml isoproterenol (cat no. I-5627, Sigma) as an internal standard for the analysis. Tissue homogenates were centrifuged at 18,000 × *g* for 10 min. The amounts of DA, DOPAC, and HVA in the supernatants were measured using a reversed-phase high-performance liquid chromatography (RP-HPLC) system (LC-10AD, Shimazu Corporation) connected to an electrochemical detector (Coulochem II, ESA). The potential electrode of the detector was set at +450 mV. Separation was performed on a reversed phase column (MCM C-18 column, 4.6 × 150 mm, MC Medical) using a single mobile phase: 50 mM acetate-citrate buffer containing 3.1% acetonitrile, 7.6% methanol, 4.4 mM sodium 1-heptanesulfonate, and 0.1 mM EDTA disodium salt, pH 3.0 at a flow rate of 1.0 ml/min in a column chamber maintained at 37°C. Data were acquired and the amount of each was calculated from peak areas using CLASS-LC10 software (Shimazu Corporation) with the standard curve of the calibration standards obtained under the same conditions.

### *In vivo* microdialysis

Mice were anesthetized with 2% vaporized isoflurane during surgery. An intracerebral guide cannula (cat no. CMA 11, CMA/Microdialysis AB) was then stereotaxically implanted into the left nucleus accumbens at a depth of 4.0 mm (coordinates with respect to the bregma: 1.4 mm anterior and 0.8 mm lateral). The guide cannula was secured with dental cement (cat no. GC Fuji I, GC Corporation) and then closed with a dummy cannula. Lidocaine cream was applied to the incision site after surgery for acute pain relief. After allowing at least 3 days recovery from surgery, the dummy cannula was removed and a dialysis probe (membrane length 1 mm, 6 kDa cut-off, CUP 11, CMA/Microdialysis AB) was set through the guide cannula. The probe was perfused with Ringer solution (147 mM Na^+^, 4 mM K^+^, and 155.6 mM Ca^2+^) at a flow rate of 2.0 μl/min. Microdialysis was performed under awake and unrestrained conditions, and the amounts of DA in dialysates were measured using online HPLC coupled to an electrochemical detector system (BMA-300, Eicom) according to the manufacturer’s instructions.

### Immunohistochemistry

Deeply anesthetized mice with isoflurane were perfused transcardially with 4% paraformaldehyde in 0.1 M phosphate buffer, pH 7.3 containing 136 mM NaCl (PBS). Brains were dissected out, incubated with 30% sucrose in PBS at 4°C overnight, and 40-μm-thick sections were cut using a cryostat. Sections were treated with 3% H_2_O_2_ and 0.05% NP-40 in TBS (50 mM Tris-HCl, pH 7.4 containing 150 mM NaCl), and then blocked with 10% normal goat serum or 4% skim milk in TBS. Sections were then incubated overnight with rabbit anti-tyrosine hydroxylase (cat no. AB152, Chemicon; 1:1000 dilution). Specific antibody binding was detected with an ABC-peroxidase kit (Vectors Laboratories) according to the manufacturer’s instructions.

### Synaptosomal preparation

Mice were habituated for at least 2 hrs to a new homecage, and this was followed by a METH or saline injection. One hour after the injection, mice were sacrificed by decapitation to avoid any complications due to anesthesia. Striatal tissues separated on an ice-cooled glass plate were pooled from 2 animals for each measurement, weighed, and homogenized in 25 (*v*/*w*) volumes of ice-cold 0.32 M sucrose with a glass-Teflon homogenizer. Striatal homogenates were centrifuged at 800×*g* for 10 min, the supernatants were centrifuged at 22,000×*g* for 15 min, and the resulting pellet (P2) was taken as the crude synaptosome fraction.

### DA uptake assay

The striatal P2 fraction was resuspended in 100 volumes (*v*/*w* original tissue weight) of modified Krebs’ buffer consisting of 16 mM phosphate buffer, pH 7.4, 126 mM NaCl, 4.8 mM KCl, 1.3 mM CaCl_2_, 1.4 mM MgSO_4_, 11 mM glucose, 1 mM ascorbic acid, and 10 μM pargyline (P8013, Sigma) purged with 95% O_2_ and 5% CO_2_. P2 suspensions were dispensed into a 96-well U-bottomed microtiter plate (50 μl per well) and preincubated at 37°C for 10 min. Assays were initiated by the addition of an equal volume of [^3^H]-DA (Amersham Pharmacia Biotech; 52.0 Ci/mmol) with unlabeled DA to obtain the final concentrations (10–640 nM), and then incubated at 37°C for 3 min. Non-specific DA uptake was assessed in the presence of 10 μM GBR12909 (D052, Sigma). Synaptosomes were then harvested onto Whatman GF/B filters presoaked with 0.05% polyethylenimine using a 96-well cell harvester. Radioactivity trapped on filters was measured using a liquid scintillation counter. Experiments were performed in triplicate for each concentration. The protein concentrations of synaptosomes were assessed with the DC Protein assay kit (Bio-rad).

### Cell surface biotinylation experiments

The striatal P2 fraction was suspended in ice cold biotinylation buffer (100 mM Triethnol amine buffer, pH 7.4, containing 1.5 M NaCl), and cell surface proteins were biotinylated using sulfo-NHS–SS–biotin (21331, Thermo Fisher Scientific) to a final concentration of 1 mg/ml on ice for 30 min. The reaction was stopped with 100 mM glycine in biotinylation buffer, and biotinylated synaptosomes were collected by centrifugation at 10,000×*g* for 5 min. Total proteins were extracted with RIPA buffer (0.5% sodium deoxycholate, 1% NP-40, and 0.1% SDS in 10 mM Tris-HCl, 150 mM NaCl, pH 7.4) containing protease inhibitors (complete, Roche). Biotinylated proteins were pulled down from the extract with streptavidin-Sepharose (GE Healthcare), and associated proteins were eluted by boiling the beads in 2×SDS-PAGE sample buffer consisting of 4% SDS and 20% glycerol in 200 mM Tris-HCl, pH 6.8.

### Western blotting

SDS-PAGE was performed on a 5–20% gradient gel (E-R520L, ATTO) and separated proteins were transferred onto a polyvinyldifluoridine membrane (Immobilon, Millipore). After blocking with 4% non-fat dry milk and 0.1% Triton X-100 in TBS, membranes were incubated with rat anti-DAT (cat no. MAB369, Millipore; 1:4000 dilution) or rabbit anti-PTPRZ-S prepared in our laboratory [19] (1:3000 dilution), followed by a treatment with HRP-conjugated anti-rabbit IgG (cat no. NA9340V, GE Healthcare; 1:3000 dilution) or anti-rat IgG (cat no. 1132-036-072, Jackson Immunoresearch; 1:4000 dilution), respectively. After washing, the blots were incubated with a chemiluminescent substrate (Luminata forte western HRP substrate, Millipore), and the chemiluminescence signal was immediately detected using a CCD camera system (Ez-capture MG, ATTO Bioscience & Technology) for a length of time sufficient to ensure detection without saturation.

### Imaging and statistical analyses

We adjusted the brightness levels of the scanned images for optimum contrast, and then obtained signal intensities using Adobe Photoshop Software (CS6 version 13.0 × 64, Adobe). Statistical analyses were performed using IBM SPSS Statistics 25 software (SPSS) together with Microsoft EXCEL (Excel for Mac version 16.16.2, Microsoft). Since ANOVA assumes the homogeneity of variance across all conditions, Mauchly’s test of Sphericity was adopted to examine the sphericity assumption within repeatedly measured data; when this result was significant, a Greenhouse-Geisser correction was applied.

## Results

### Increased behavioral responses to novelty in *Ptprz-KO* mice

**Fig 1:**
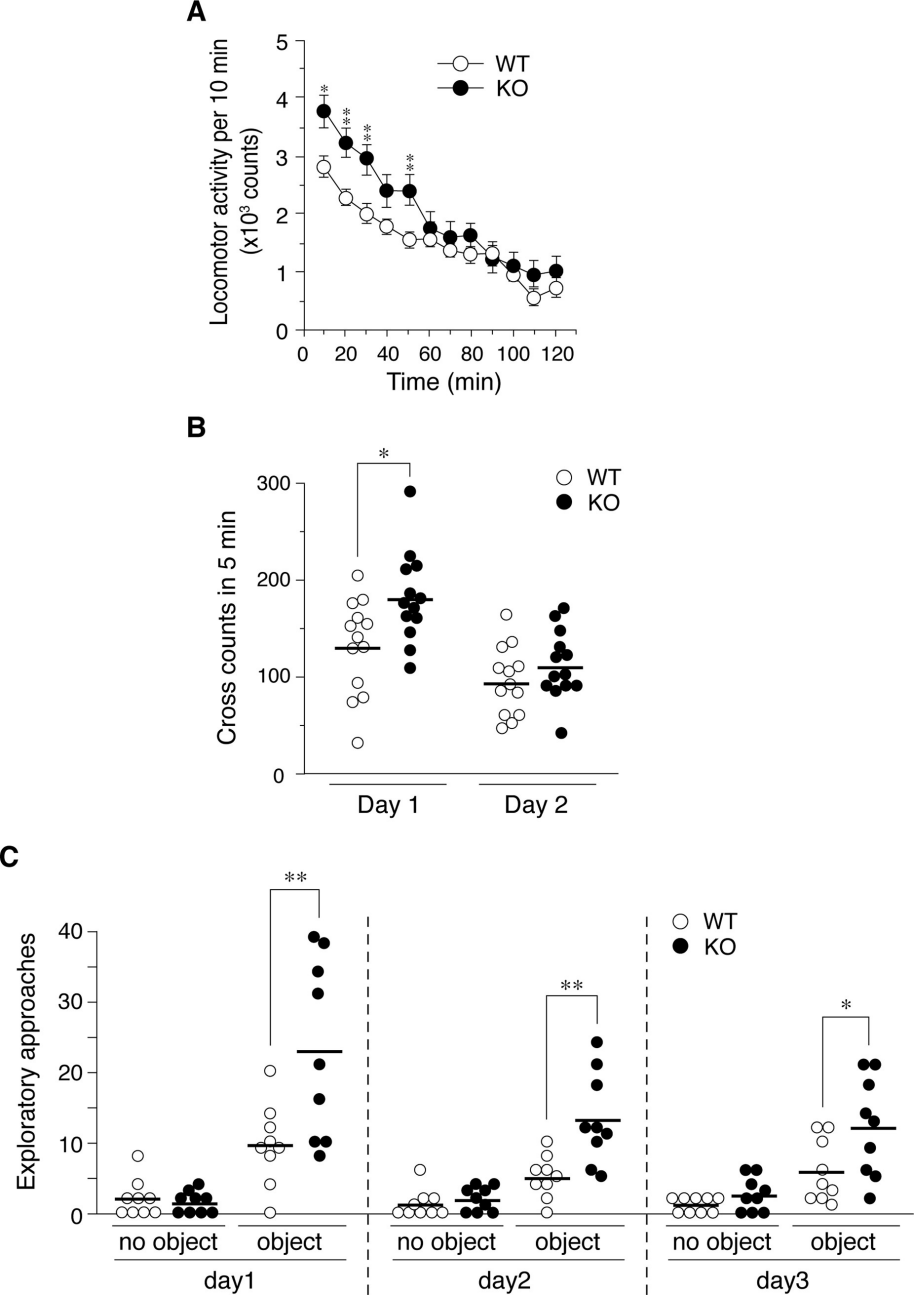
Increases in novelty responses in *Ptprz*-KO mice. (**A**) Locomotor activity. The locomotor activities of wild-type (WT) and *Ptprz*-KO (KO) were automatically measured as indexed by the number of sensor counts at 10-min intervals. The graph shows mean values with the standard error (SE) for each group (*n* = 13 per group). There were significant main effects of time (*F*_(4.962, 119.081)_ = 45.860, *P* = 0.000, ε = 0.451), and genotype (*F*_(1, 24)_ = 4.291, *P* = 0.049), and a significant interaction between time and genotype (*F*_(4.962, 119.081)_ = 3.074, *P* = 0.012, ε = 0.451) by a two-way mixed design ANOVA. *, *P* < 0.05; **, *P* < 0.01, significantly different from wild-type mice by Bonferroni’s *post-hoc* test. (**B**) Horizontal activity in the open field. Tests were performed for 5 min per day on 2 consecutive days. The scatter plot shows individual values of locomotor activity with a horizontal bar designating the mean (*n* = 13 per group). There were significant main effects of day (*F*_(1, 24)_ = 42.074, *P* = 0.000), genotype (*F*_(1, 24)_ = 5.375, *P* = 0.029), and a significant interaction between day and genotype (*F*_(1, 24)_ = 4.274, *P* = 0.05) by a two-way mixed design ANOVA. *, *P* < 0.05, significantly different from wild-type mice by Bonferroni’s *post-hoc* test. (**C**) Novel object exploration test. Tests were performed in the same open field, first in the absence and then in the presence of an object for 9 min each on 3 consecutive days. The scatter plot shows individual values of the exploratory activity with their mean (*n* = 9 per group). There were significant main effects of object (Day 1, *F*_(1, 16)_ = 35.631, *P* = 0.000; Day 2, *F*_(1, 16)_ = 56.006, *P* = 0.000; Day 3, *F*_(1, 16)_ = 26.089, *P* = 0.000) and genotype on all three days (Day 1, *F*_(1, 16)_ = 7.867, *P* = 0.013; Day 2, *F*_(1, 16)_ = 8.967, *P* = 0.009; Day 3, *F*_(1, 16)_ = 6.590, *P* = 0.021) by a two-way mixed design ANOVA. *, *P* < 0.05; **, *P* < 0.01, significantly different between indicated groups by Bonferroni’s *post-hoc* test.

When mice were placed in an ambulation chamber, *Ptprz*-KO mice were significantly more active than wild-type mice for ~50 min after exposure to the novel environment; however, their activity subsequently returned to similar basal level between the two groups (Fig 1A). *Ptprz*-KO mice also showed significantly higher horizontal activity than wild-type mice in a novel inescapable open-field test on day 1, and this difference disappeared on day 2 with habituation (Fig 1B).

We then examined responses to a novel object in the habituated open field. Both groups of mice rarely moved into the central region of the open field with no objects. After the introduction of a novel object at the center, mice began to visit the central zone to explore the object, with approach frequency to the object being significantly higher in *Ptprz*-KO than in wild-type mice on day 1 (Fig 1C). Exploratory behavior gradually decreased because of habituation to the object. The increase observed in the behavioral response to novelty in *Ptprz*-KO was not considered to be due to a decrease in anxiety because no significant differences were observed between the two genotypes in the elevated-plus maze test of anxiety previously [20].

### No gross morphological abnormalities in the *Ptprz*-KO mouse brain

**Fig 2:**
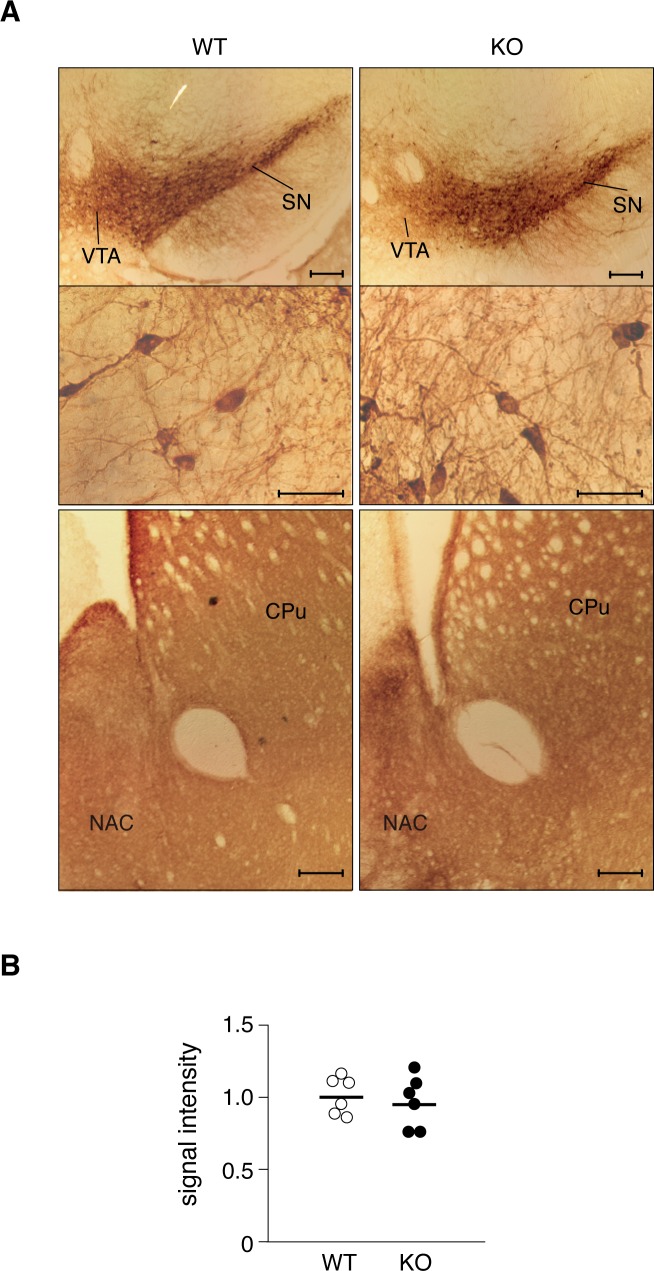
No histological changes in dopaminergic pathways in the brain of *Ptprz*-KO mice. **(A)** Immunohistochemistry of coronal brain sections by anti-tyrosine hydroxylase. Images are the midbrain region with low- (top panels) and high- (middle panels) magnification, and striatum (bottom panels). Scale bars, 500 μm (top and bottom panels) and 50 μm (middle panels). CPu, caudate putamen; NAc, nucleus accumbens; SN, substantia nigra; and VTA, ventral tegmental area. Results are representative of six individual mice. (**B**) Comparison of the signal intensity of anti-TH staining in the striatum. The scatter plot shows individual values of the signal intensity relative to that of averaged values of wild-type mice (*n* = 6 per group). There were no significant differences between the genotypes (*P* = 0.480 by the Student’s *t*-test).

*Ptprz*-KO mice show no gross morphological abnormalities in the brain [15]. Nevertheless, a subsequent study suggested the possibility that PTPRZ-S is involved in the lateral migration of mesencephalic DA neurons during development [21]. However, our present immunohistochemical analysis revealed that the innervation patterns of the mesolimbic and mesostriatal dopaminergic pathways were indistinguishable between wild-type and *Ptprz*-KO mice (Fig 2). We also examined the activity of tyrosine hydroxylase (TH), the rate-limiting enzyme for DA biosynthesis, using brain extracts, together with other neurotransmitter synthesizing enzymes, such as glutamate decarboxylase (GAD) for γ-aminobutyric acid (GABA) biosynthesis and choline acetyltransferase for acetylcholine (Ach) biosynthesis. However, these enzyme activities did not significantly differ between the two genotypes (S1 Table).

**Fig 3:**
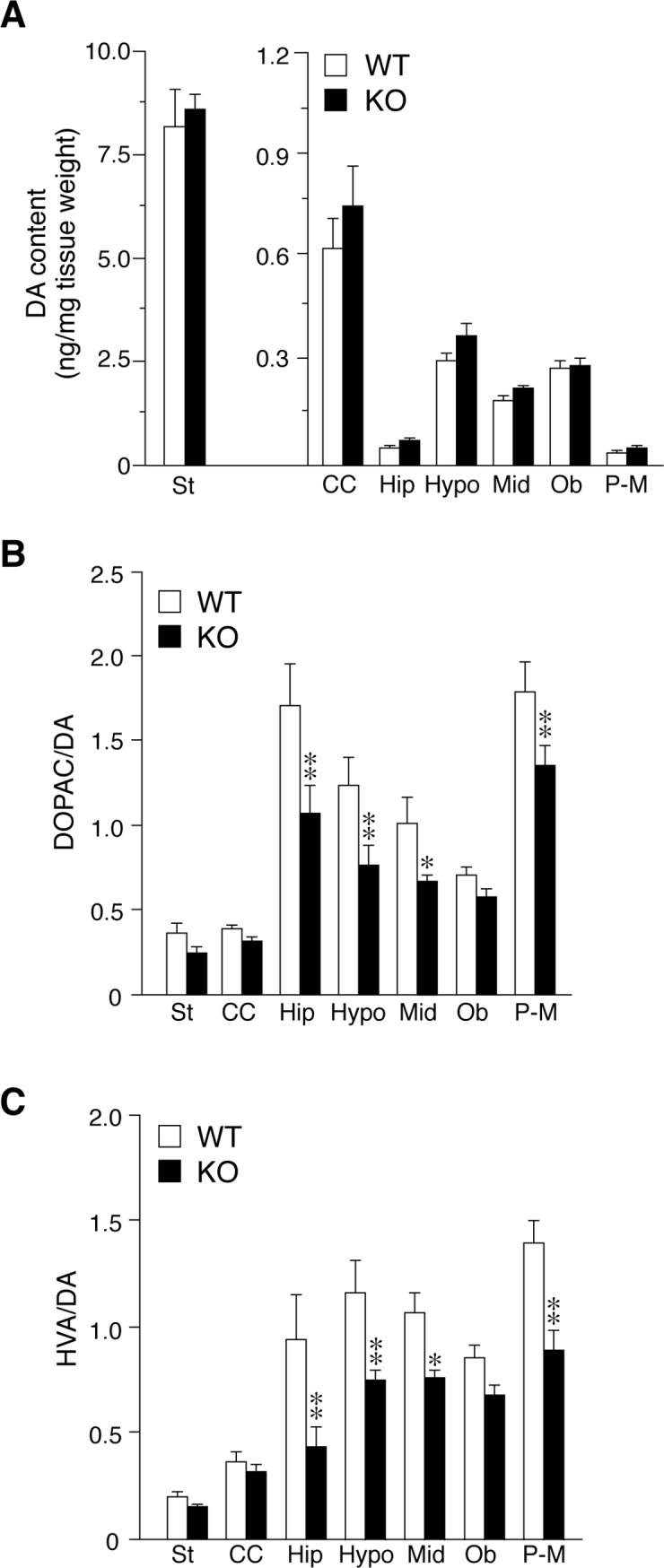
Changes in DA content and its turnover in brain tissues of *Ptprz-*KO mice. (**A-C**) Measurements of tissue DA content, and its metabolites DOPAC and HVA in different brain regions. DA content (*A*), and DOPAC/DA (*B*) and HVA/DA (*C*) ratios, which are regarded as index values of dopamine turnover (*n* = 12–13 per group). Graphs show mean values with SE. There were significant main effects of genotype on DOPAC/DA and HVA/DA (DA, *F*_(1, 165)_ = 0.567, *P* = 0.452; DOPAC/DA, *F*_(1, 165)_ = 22.539, *P* = 0.000; and HVA/DA, *F*_(1, 165)_ = 31.840, *P* = 0.000) and tissue region on all groups (DA, *F*_(6, 165)_ = 295.197, *P* = 0.000; DOPAC/DA, *F*_(6, 165)_ = 31.058, *P* = 0.000; and HVA/DA, *F*_(6, 165)_ = 26.735, *P* = 0.000), and significant interactions between genotype and tissue region on HVA/DA (DA, *F*_(6, 165)_ = 0.187, *P* = 0.980; DOPAC/DA, *F*_(6, 165)_ = 1.512, *P* = 0.177; and HVA/DA, *F*_(6, 165)_ = 2.273, *P* = 0.039) by a two-way ANOVA. *, *P* < 0.05; **, *P* < 0.01, significantly different from wild-type mice in the same region by Bonferroni’s *post-hoc* test. St, Striatum; CC, Cerebral Cortex; Hip, Hippocampus; Hypo, Hypothalamus; Mid, Mesencephalon; Ob, Olfactory bulb; and P-M, Pons and Medulla.

In brain tissues, neurons and glial cells both express PTPRZ [15]. We found that >80% midbrain DA neurons expressed PTPRZ receptor isoforms using single cell RT-PCR (S1 Fig), and that PTPRZ and DAT colocalized well in primary cultured DA neurons (S2 Fig). Neurochemical analyses revealed that DA contents in brain tissues were similar between the two genotypes (Fig 3A); however, the turnover rate, expressed as a ratio of its metabolite, dihydroxyphenylacetic acid (DOPAC) or homovanillic acid (HVA), to DA was significantly lower in various brain regions (except for the striatum and cerebral cortex) in *Ptprz-*KO mice than in wild-type mice (Fig 3B and 3C). DAT activity is functionally related to DA turnover [22]. Together, these results suggested that a PTPRZ deficiency may cause functional alterations, such as reductions in METH-induced and DAT-mediated DA release and DA turnover, and therefore we focused on the effect of *Ptprz* deficiency on DAT functions.

### Reduced dopaminergic responses to METH in *Ptprz-KO* mice

**Fig 4:**
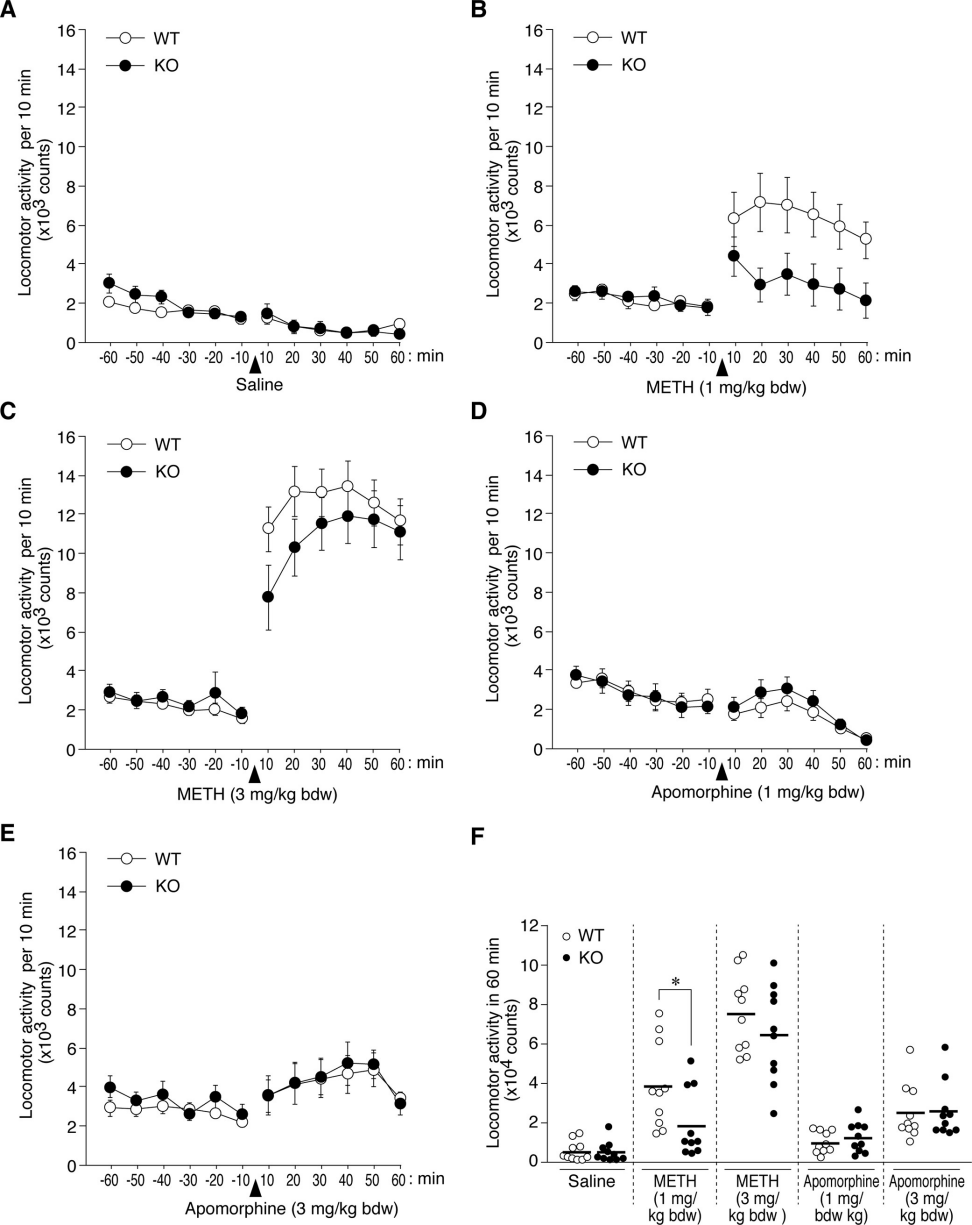
Decreases in locomotor responses to a single METH injection in *Ptprz*-KO mice. (**A-F**) Time course of locomotor activity. Mice were habituated to the ambulation chamber for at least 80 min before drug administration, followed by a subcutaneous (sc) injection as indicated in the figures, and set as time zero. Graphs show the mean values with SE for each group at each time point (*n* = 10 per group). The results of a two-way mixed design ANOVA (main effects of time and genotype, and an interaction effect between time and genotype) for each group after treatment (10 to 60 min) were as follows: METH at 1 mg/kg bdw (*F*_(2.147, 38.644)_ = 3.248, *P* = 0.046, ε = 0.429; *F*_(1, 18)_ = 4.855, *P* = 0.041; and *F*_(2.147, 38.644)_ = 1.204, *P* = 0.313, ε = 0.429), METH at 3 mg/kg bdw (*F*_(1.729, 31.122)_ = 5.108, *P* = 0.015, ε = 0.346; *F*_(1, 18)_ = 1.196, *P* = 0.289; and *F*_(1.729, 31.122)_ = 1.320, *P* = 0.279, ε = 0.346), apomorphine at 1 mg/kg bdw (*F*_(1.853, 33.354)_ = 16.960, *P* = 0.000, ε = 0.371; *F*_(1, 18)_ = 0.656, *P* = 0.428; and *F*_(1.853, 33.354)_ = 0.562, *P* = 0.562, ε = 0.371), apomorphine at 3 mg/kg bdw (*F*_(2.003, 36.051)_ = 3.480, *P* = 0.041, ε = 0.401; *F*_(1, 18)_ = 0.011, *P* = 0.918; and *F*_(2.003, 36.051)_ = 0.143, *P* = 0.867, ε = 0.401), and saline (*F*_(2.456, 44.217)_ = 9.855, *P* = 0.000, ε = 0.491; *F*_(1, 18)_ = 0.022, *P* = 0.884; and *F*_(2.456, 44.217)_ = 1.406, *P* = 0.256, ε = 0.491). (**F**) Summary of locomotor activity. The scatter plot shows individual values of locomotor activity with their mean for 60 min after the drug injection. *P < 0.05, significantly different between the indicated pair by the Student’s *t*-test.

We tested the effects of METH, a DA releaser/DAT inhibitor, on locomotor activity at non-toxic low doses (less than 4 mg or 10 mg per kg bdw in C57BL/6 mice [23] or rats [24], respectively) to avoid the behavioral stereotypy known to accompany higher doses. METH-induced increases in locomotion were observed in *Ptprz*-KO and wild-type mice relative to saline administration; however, the degree of this increase was significantly smaller at a dose of 1 mg per kg bdw in *Ptprz*-KO mice than in wild-type mice (Fig 4A, 4B and 4F). METH-induced locomotor activity was further potentiated at a dose of 3 mg per kg bdw, although the difference between the two genotypes disappeared (Fig 4C and 4F). When injected with a non-selective DA receptor agonist, apomorphine (1 or 3 mg per kg bdw), mice in both groups showed almost no hyperlocomotion (Fig 4D to 4F). Repetitive stereotyped behaviors, such as grooming and sniffing, were observed in both groups, though we could not analyze the differences quantitatively, because our monitoring apparatus was not equipped with a video camera. Nevertheless, this result suggested that the difference in METH-induced hyperactivity was not due to changes in the DA receptor itself.

**Fig 5:**
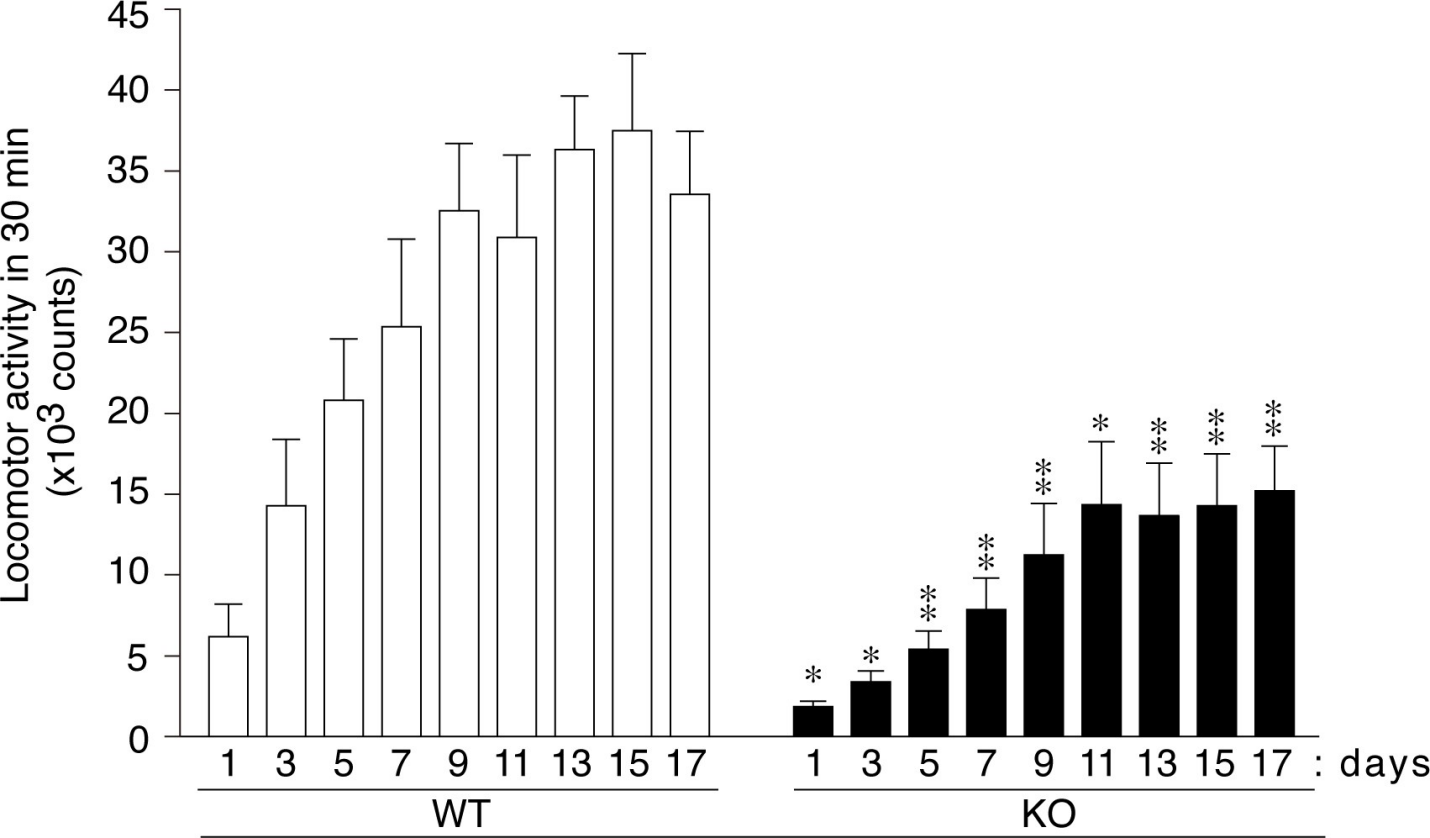
No changes in locomotor sensitization by repeated METH administration in *Ptprz*-KO mice. Mice treated daily with a sc injection of METH at 1 mg per kg bdw in an ambulation chamber for 17 consecutive days. Graphs show the mean of locomotor activity for 60 min with SE following the drug injection (*n* = 9–10 per group). There were significant effects of day (*F*_(4.106, 69.802)_ = 22.933, *P* = 0.000, ε = 0.513), genotype (*F*_(1, 17)_ = 18.450, *P* = 0.000), and an interaction between day and genotype (*F*_(4.106, 69.802)_ = 3.368, *P* = 0.013, ε = 0.513) by a two-way mixed design ANOVA. *, *P* < 0.05; **, *P* < 0.01, significantly different from wild-type mice on the same day by Bonferroni’s *post-hoc* test.

Furthermore, the repeated administration of METH at a dose of 1 mg per kg bdw per day induced progressively increased locomotion (locomotor sensitization), which reached constant values by day 11 in both genotypes; however, the maximum activity level in *Ptprz*-KO mice remained lower than that in wild-type mice (Fig 5). These results indicated reduced, but not impaired, METH responsiveness in *Ptprz*-KO mice.

### Reduced METH-induced DA release in *Ptprz*-KO mice

**Fig 6:**
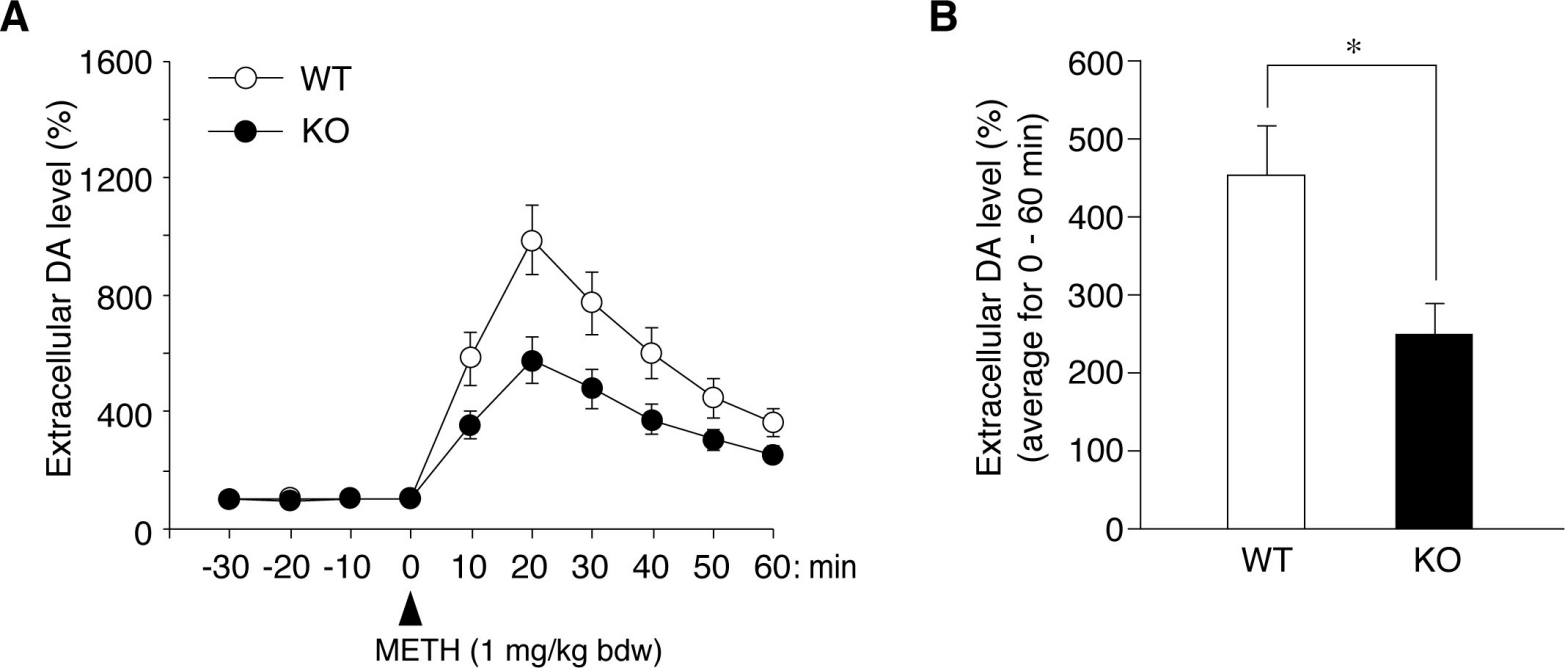
Decrease in METH-evoked DA release in the nucleus accumbens in *Ptprz*-KO mice. (**A, B**) Microdialysis measurements of extracellular DA levels in the nucleus accumbens in freely moving mice. The amount of DA in perfusates was automatically analyzed using an on-line HPLC system at 10-min intervals. The plot shows the mean ± SE of the DA levels in perfusates (*n* = 10 per group) (*A*). After the stabilization of basal DA levels in perfusates, mice were administered METH at 1 mg per kg bdw and set as time zero. DA levels were expressed as a percentage of averaged basal values (three points, -30 ~ -20 min to -10 ~ 0 min). The basal DA level (the mean ± standard deviation given as artificial units) were 21.9 ± 10.4 in WT and 23.0 ± 18.3 in *Ptprz*-KO perfusates, respectively, and there were no significant differences (*P* = 0.872) between the genotypes by the Student’s *t*-test. (**B**) Summary of METH-evoked DA release. The graph shows the mean with SE of extracellular DA levels for 60 min after the METH treatment. *, *P* < 0.05, significantly different from wild-type mice by the Student’s *t*-test.

METH is known to increase extracellular DA levels in the nucleus accumbens by facilitating its efflux through DAT, thereby inducing hyperlocomotion [1]. Microdialysis measurements revealed that METH-evoked DA release in the nucleus accumbens was significantly lower in *Ptprz*-KO mice than in wild-type mice, without changing basal extracellular DA levels (Fig 6). However, depolarization-evoked DA release was similar between the two genotypes (S3 Fig).

### Decreased DA uptake activity in METH-treated *Ptprz-*KO mice

**Fig 7:**
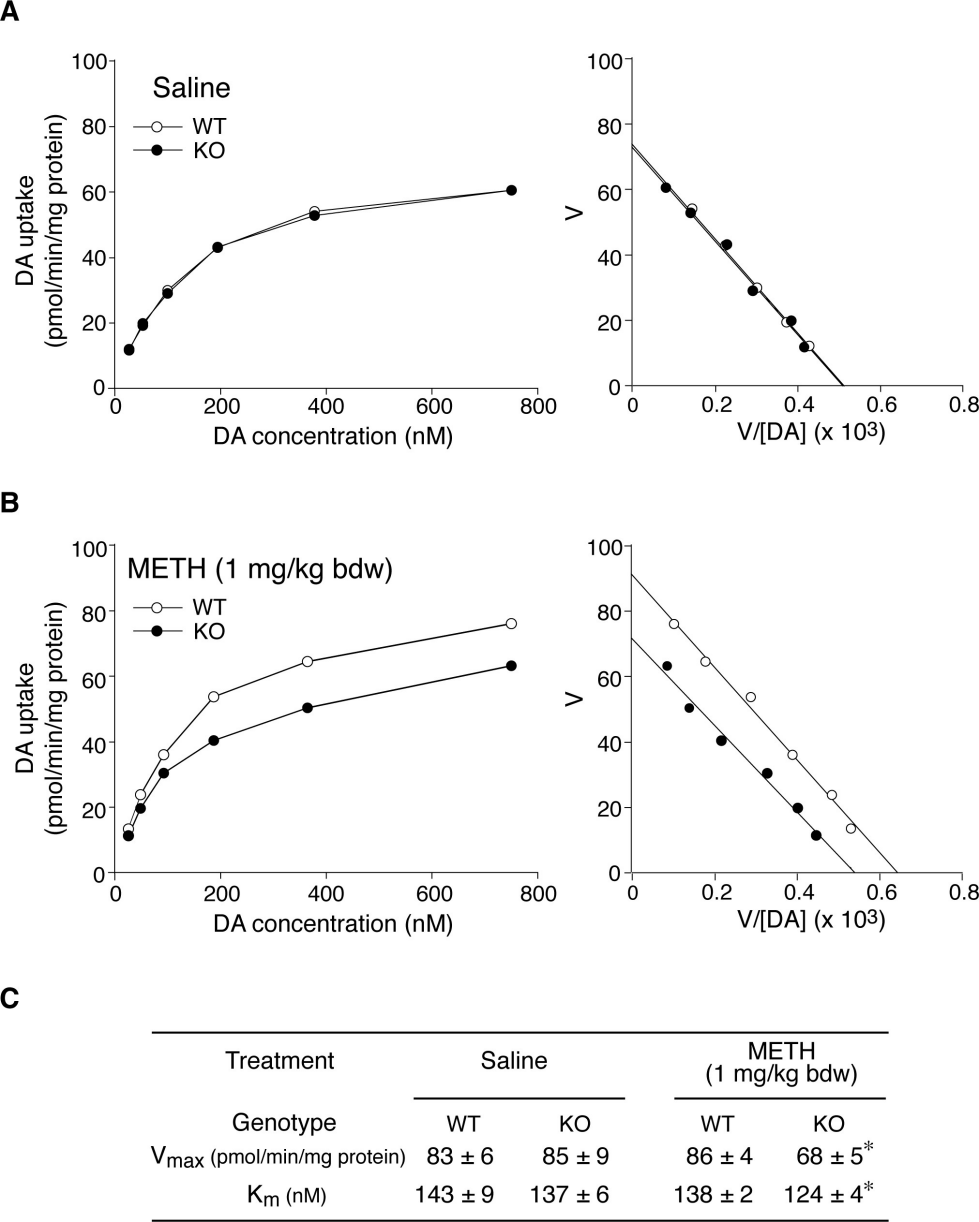
Decrease in DA uptake activity in striatal synaptosomes of *Ptprz-*KO mice. (**A, B**) Saturation curves (left) and Eadie-Hofstee plots (right) of DA uptake in striatal synaptosomes. DA uptake assays were performed on striatal synaptosomes prepared from mice 1 hr after the sc injection of saline (A) or METH at 1 mg per kg bdw (B), in which four striata from two mice were pooled as a sample per treatment group. Each sample was assayed in triplicate for each concentration, and the averaged values were subjected to Eadie-Hofstee analysis to determine K_m_ and V_max_. One representative set of results out of three independent experiments is shown. (**C**) Summary of DA uptake experiments. K_m_ and V_max_ values obtained from three independent experiments are shown as the mean ± standard deviation. *, *P* < 0.05, significantly different from the wild-type with the same treatment by the Student’s *t*-test.

A previous study by Fleckenstein et al. indicated that a single METH injection at doses of 5 mg per kg bdw or higher caused significant decreases in DA uptake by striatal synaptosomes prepared from rats 1 hr after the administration of METH [25]. DA uptake is solely mediated by DAT on the cell surface. When synaptosomes were prepared from mice 1 hr after the administration of saline (control), wild-type and *Ptprz*-KO mice showed very similar DA uptake activities (Fig 7A), suggesting normal DAT activity under basal conditions. However, in synaptosomes prepared from mice 1 hr after the administration of METH, DA uptake in *Ptprz*-KO appeared to be reduced (Fig 7B; ~20% decrease in Vmax values and ~10% increase in Km values, see Fig 7C). In contrast, a similar change was not observed in wild-type mice, which was consistent with the findings of the rat study [25].

**Fig 8:**
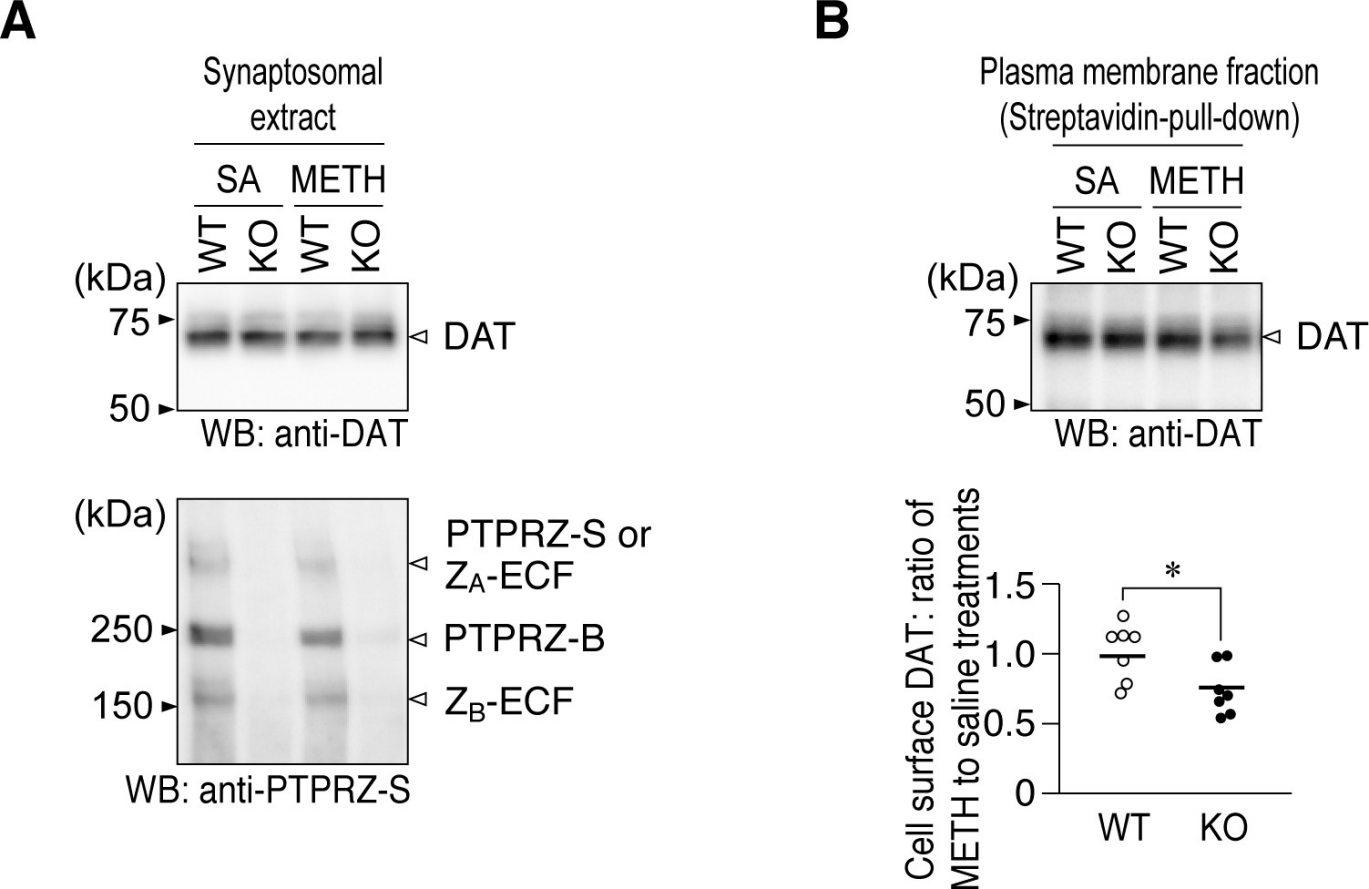
Cell surface levels of DAT proteins in striatal synaptosomes of *Ptprz-*KO mice. (**A**, **B**) Cell-surface biotinylation experiments. Striatal synaptosomes were prepared from mice 1 hr after the injection of METH at 1 mg per kg bdw or saline (SA), with four striata from two mice being pooled per experiment. The cell surface proteins of striatal synaptosomes were biotinylated, and biotinylated proteins were then recovered with streptavidin beads. Total protein levels in synaptosomes (*A*) and streptavidin isolates (*B*) were analyzed by Western blotting using the indicated antibodies (full-length blots are shown in S4 Fig.). The scatter plot shows the signal intensity of DAT staining in the streptavidin pull-down fraction from METH-treated mice relative to that from saline-treated mice of the same genotype, in which each circle corresponds to an independent experiment (*n* = 7 per group). *, *P* < 0.05, significantly different from wild-type mice by the Student’s *t*-test. Three PTPRZ isoforms (PTPRZ-A, -B, and -S) and their processed derivatives (Z_A_-ECF and Z_B_-ECF) were detected in the brain [26, 19]. This result suggested that a PTPRZ deficiency might lead to increased METH-induced sequestration or loss of DAT proteins from the cell surface, which constrained the activity of DAT activity.

To test whether the decrease in DA uptake resulted from a change in cell surface DAT proteins (sequestration), we performed cell surface biotinylation experiments using synaptosome fractions from wild-type and *Ptprz*-KO mice treated with saline or METH. Western blots of synaptosome extracts showed that the total amount of DAT proteins was similar in all four groups, and the core proteins of the short receptor PTPRZ-B isoform were predominantly detected in wild-type samples (Fig 8A). METH-treated *Ptprz*-KO mice showed a significant reduction in DAT levels in the streptavidin pull-down fraction as compared with saline-treated mice (Fig 8B) without changes in the total amount of DAT proteins (Fig 8A). On the other hand, the same METH treatment did not induce the change in wild-type mice (Fig 8B). These results suggested that a PTPRZ deficiency may lead to an increased METH-induced sequestration of DAT proteins from the cell surface, which constrained the activity of DAT activity.

## Discussion

High novelty seekers are commonly considered to be at higher risk of using drugs of abuse, including METH and AMPH, than low novelty seekers because exposure to novelty activates the same neural substrate that mediates the rewarding effects of drugs of abuse [4, 5]. In experimental animals, METH is also known to produce more potent stimulant effects in high novelty responders than in low responders [27]. Contrary to these notions, the present results indicate that *Ptprz*-KO mice are high novelty responders, but associated with a low METH response. Although novelty seeking as well as drug seeking behaviors activate the mesolimbic DA system [5], a number of other brain structures are also considered to play a role in novelty seeking behavior, including the hippocampus [28] which contributes to novelty detection [29, 30]; the hippocampus may compute a novelty signal that is subsequently propagated to the nucleus accumbens. It is important to note that PTPRZ is widely and abundantly expressed in the hippocampus in adult mice. *Ptprz*-KO mice exhibited a maturation-dependent alteration in long-term potentiation (LTP) in the CA1 region in hippocampal slices [31], which was associated with impairments in learning and memory, as evidenced by significantly prolonged latency in the hidden platform tasks of the Morris water maze [31] and by reduced freezing behaviors in the contextual fear conditioning [20], suggesting that the high novelty response observed in *Ptprz*-KO mice may rather reflect hippocampal-dependent learning impairments than dopaminergic dysfunctions.

*Ptprz*-KO mice show no histological abnormalities in the mesolimbic or mesostriatal dopaminergic pathways, which is consistent with findings obtained in another *Ptprz*-knockout mouse strain [32]. Reduced METH responsiveness may be explained by the higher sequestration of cell surface DAT proteins induced by the administration of METH. The *Ptprz* gene encodes three major splicing isoforms: Two transmembrane receptor isoforms, PTPRZ-A and PTPRZ-B, and one secretory isoform, PTPRZ-S, and neither of them is expressed in *Ptprz*-KO mice [15, 19, 33, 34]. The single-cell RT-PCR experiments revealed that the majority of midbrain DA neurons express PTPRZ receptor isoforms, and the core proteins of the PTPRZ-B receptor were found to be enriched in striatal synaptosome fractions of WT mice, suggesting that the loss of PTPRZ receptor functions may induce dopaminergic phenotypes in *Ptprz*-KO mice. We very recently found that phosphatase-inactive PTPRZ mutant (*Ptprz*-CS) mice generated by mutagenesis of the key cysteine residue to serine in the active center also exhibited attenuated locomotor activations by METH with reductions in DA efflux in the nucleus accumbens [35]. On the other hand, increased responses to novelty and impaired memory retention in the aversive learning task were observed in Ptprz-KO mice, but not in Ptprz-CS mice [35]. These results suggested that low METH responsiveness is due to the loss of the PTPase activity of PTPRZ-A/B receptor isoforms, whereas high novelty response may be related to impaired memory retention by the loss of the secretory isoform, PTPRZ-S, probably in the hippocampus [20, 31]: It is therefore considered that the high novelty response and decreased METH response are based on independent molecular mechanisms.

The phosphorylation of DAT at tyrosine residues has not been detected by ^32^PO_4_ labeling or anti-phosphotyrosine immunoblotting [36], which excludes the possibility of DAT being a direct substrate of PTPRZ. On the other hand, a recent study indicated that the glial cell line-derived neurotrophic factor (GDNF) and its canonical receptor RET tyrosine kinase regulate the surface expression of DAT through a signaling cascade that involves the Rho-family guanine nucleotide exchange factor protein, VAV2 [10, 37]. Mice deficient in RET exhibited elevated DAT activity in the nucleus accumbens, but showed reduced DAT activity associated with diminished behavioral responses to cocaine, suggesting that GDNF/RET signaling is a key determinant of DAT trafficking *in vivo* [10]. The loss of one or some PTPs, which counteract GDNF-RET signaling, may cause a reduction in METH responsiveness through DAT regulation, though the typical PTPRZ substrate motif [38] was not found in the sequence of RET or VAV2. Further studies are needed to assess the involvement of the PTPase activity of PTPRZ and its physiological substrate molecules [38–44] in the regulation of DAT trafficking in mesolimbic DA neurons.

## Supporting information

S1 Table No changes in enzyme activities of DA, GABA, and Ach biosynthesis in different brain tissues. (TIF)

S1 Fig Single cell RT-PCR analyses. (**A**) Primer sequences used for single-cell RT-PCR. Target genes (and their relative GeneBank accession number) and multiplex and nested primer sets (and their position) are listed. Tyrosine hydroxylase, (TH) as a DA neuron marker; glutamate decarboxylase 1, (Gad1) as a GABA neuron marker, and glial fibrillary acidic protein, (Gfap) as an astrocyte marker. Regarding PTPRZ, primer sets were designed to target a part of the intracellular region. The specific amplified products by second nested PCR for *Ptprz*, *Th*, *Gad1*, and *Gfap* are 189, 377, 702, and 517 bp, respectively. (**B**) Results of single-cell RT-PCR analyses. Typical single-cell PCR patterns identified as DA neurons (*Th*^+^) and GABA neurons (*Gad1*^+^) are shown. Positive control (mouse brain cDNA) and negative control (mouse genomic DNA) experiments are shown. Control PCR detected each of the specific products from mouse brain cDNA, whereas no bands were detected with mouse genomic DNA. We prepared cDNA from 50 single cells derived from the substantia nigra and ventral tegmental area separated from four individual wild-type mouse brains, and identified 28 DA neurons (*Th*^+^, *Gad1*^-^, *Gfap*^-^) and 6 GABA neurons (*Th*^-^, *Gad1*^+^, *Gfap*^-^), in which *Ptprz* was amplified in 82% (23/28) and 50% (3/6) of cells, respectively.
(TIF)

S2 Fig Colocalization of PTPRZ and DAT in DA neurons. Immunofluorescence microscopy of primary cultured dopaminergic neurons. Double labeling with the anti-PTPRZ (red) and anti-DAT (green) antibodies revealed that PTPRZ proteins colocalized with DAT proteins.
(TIF)

S3 Fig High K+-evoked DA release in the nucleus accumbens. (**A, B**) Microdialysis measurements were performed as shown in Fig 6. Plots show the mean ± SE of wild-type and *Ptprz*-KO (*n* = 9–10 per group) (*A*). The horizontal bar indicates the application of 100 mM K^+^ solution (for 20 min) via the microdialysis probe. Dopamine levels were expressed as a percentage of averaged basal values (two points, -40 ~ -20 min and -20 ~ 0 min). Summary (*B*). The bar graph shows the mean with SE of extracellular DA levels for 60 min after the application of high K^+^ solution. There was no significant difference between the two genotypes (the Student’s *t*-test).
(TIF)

S4 Fig Full-length blots for Fig 8. (TIF)

S1 Text Supplementary materials and methods. (DOCX)

## Abbreviations

AMPH: Amphetamine
DA: Dopamine
DAT: DA transporter
DOPAC: Dihydroxyphenylacetic acid
GFAP: Glial l fibrillary acidic protein
GDNF: Glial cell line-derived neurotrophic factor
GAD: Glutamate decarboxylase
HVA: Homovanillic acid
6-OHDA: 6- Hydroxydopamine
KO: Knockout
LTP: Long-term potentiation
METH: Methamphetamine
MMP: Matrix metalloproteinase
PTKs: Protein tyrosine kinases
PTPs: Protein tyrosine phosphatases
PTPRZ: Protein tyrosine phosphatase receptor type Z
RPTPs: Receptor-like protein tyrosine phosphatases
Vav2: Rho-family guanine nucleotide exchange factor protein
tPA: Tissue plasminogen activator
TH: Tyrosine hydroxylase
