# Supplementary figures and images for: Mice deficient in protein tyrosine phosphatase receptor type Z (PTPRZ) show reduced responsivity to methamphetamine despite an enhanced response to novelty
Source: PLoS One. 2019 Aug 20;14(8):e0221205. doi: 10.1371/journal.pone.0221205 (PMC6701799; doi:10.1371/journal.pone.0221205)

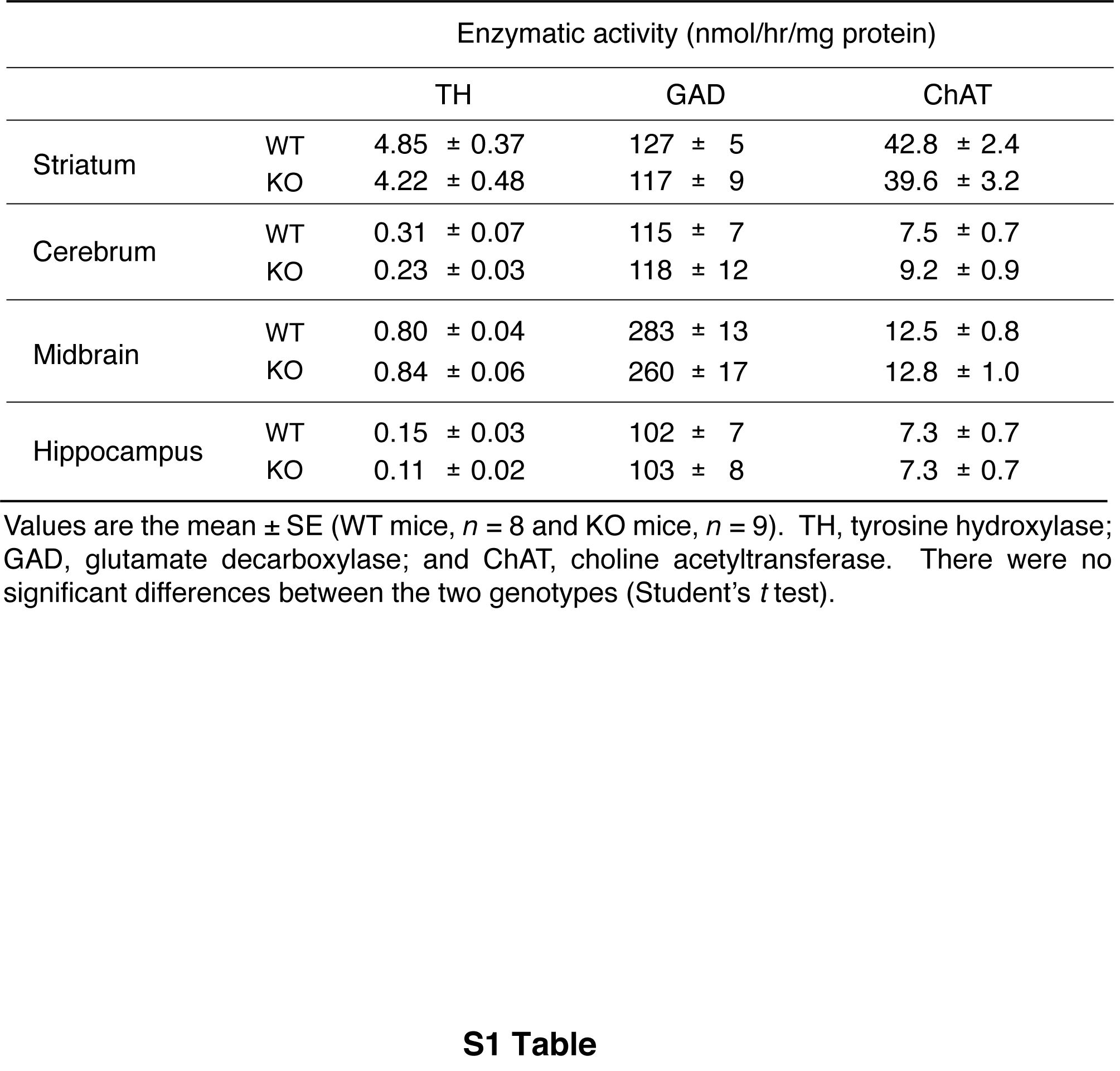

Supplement: S1 Table — (TIF) [file pone.0221205.s001.tif]

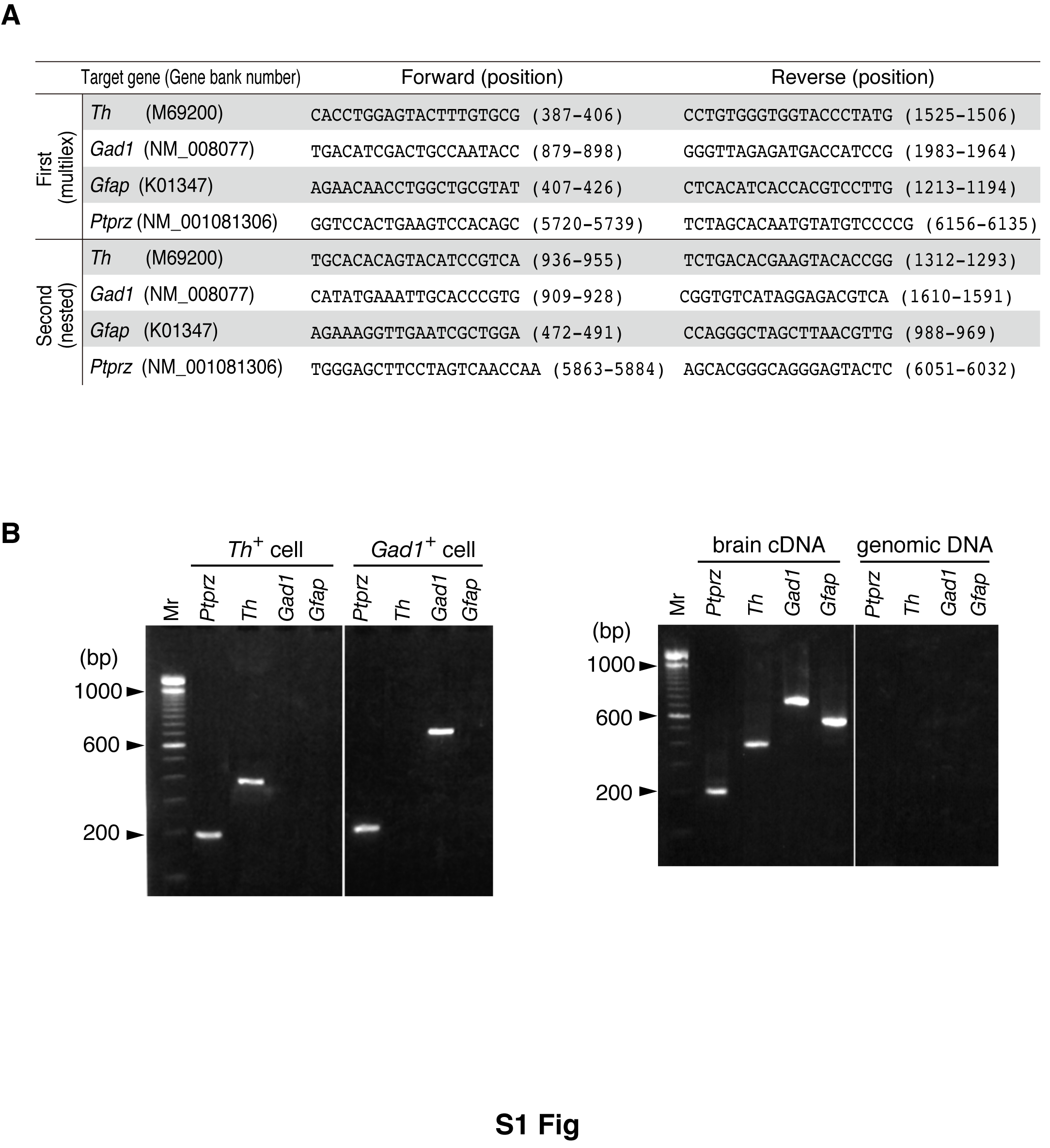

Supplement: S1 Fig — (A) Primer sequences used for single-cell RT-PCR. Target genes (and their relative GeneBank accession number) and multiplex and nested primer sets (and their position) are listed. Tyrosine hydroxylase, (TH) as a DA neuron marker; glutamate decarboxylase 1, (Gad1) as a GABA neuron marker, and glial fibrillary acidic protein, (Gfap) as an astrocyte marker. Regarding PTPRZ, primer sets were designed to target a part of the intracellular region. The specific amplified products by second nested PCR for Ptprz, Th, Gad1, and Gfap are 189, 377, 702, and 517 bp, respectively. (B) Results of single-cell RT-PCR analyses. Typical single-cell PCR patterns identified as DA neurons (Th+) and GABA neurons (Gad1+) are shown. Positive control (mouse brain cDNA) and negative control (mouse genomic DNA) experiments are shown. Control PCR detected each of the specific products from mouse brain cDNA, whereas no bands were detected with mouse genomic DNA. We prepared cDNA from 50 single cells derived from the substantia nigra and ventral tegmental area separated from four individual wild-type mouse brains, and identified 28 DA neurons (Th+, Gad1-, Gfap-) and 6 GABA neurons (Th-, Gad1+, Gfap-), in which Ptprz was amplified in 82% (23/28) and 50% (3/6) of cells, respectively. (TIF) [file pone.0221205.s002.tif]

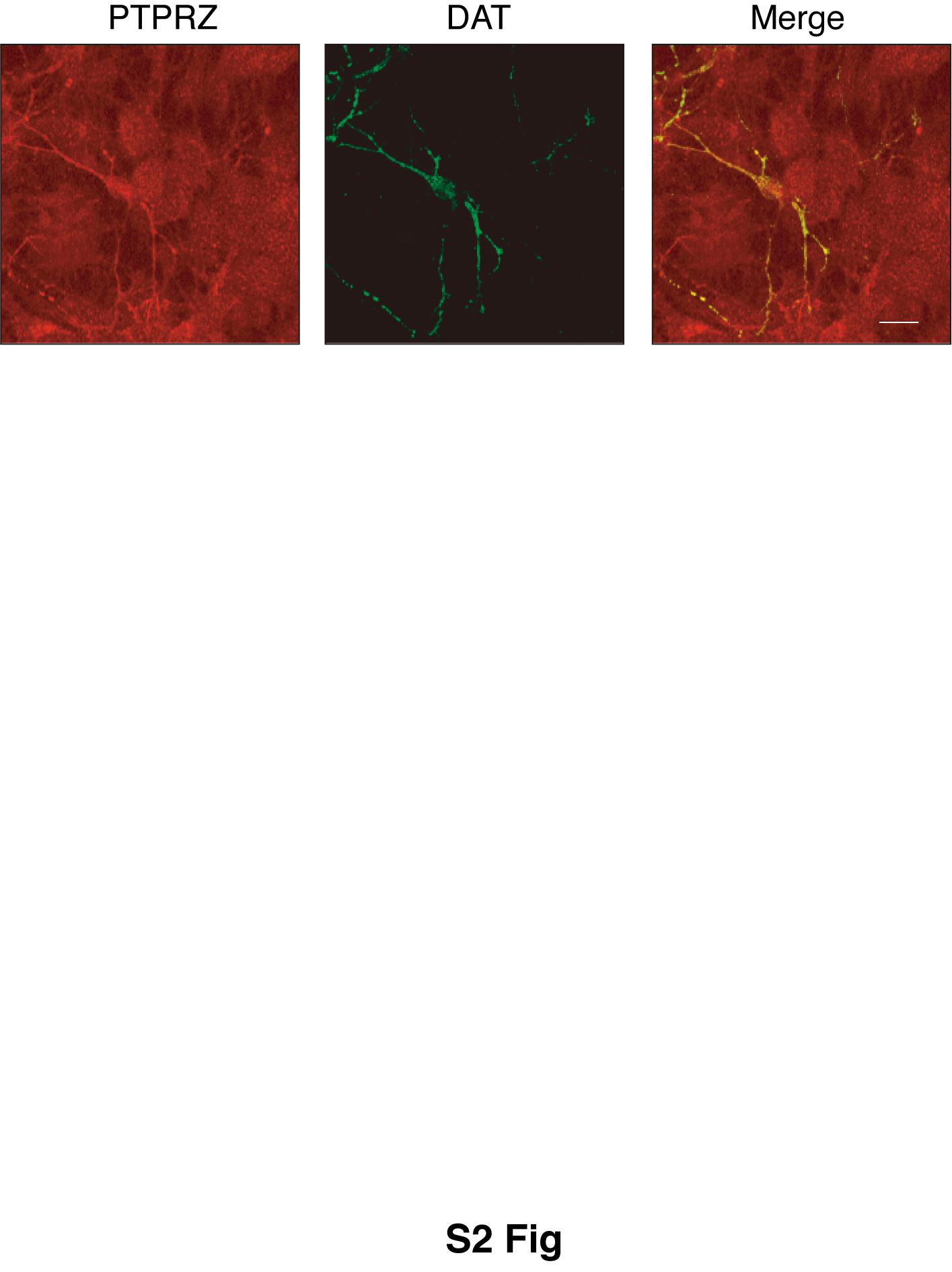

Supplement: S2 Fig — Immunofluorescence microscopy of primary cultured dopaminergic neurons. Double labeling with the anti-PTPRZ (red) and anti-DAT (green) antibodies revealed that PTPRZ proteins colocalized with DAT proteins. (TIF) [file pone.0221205.s003.tif]

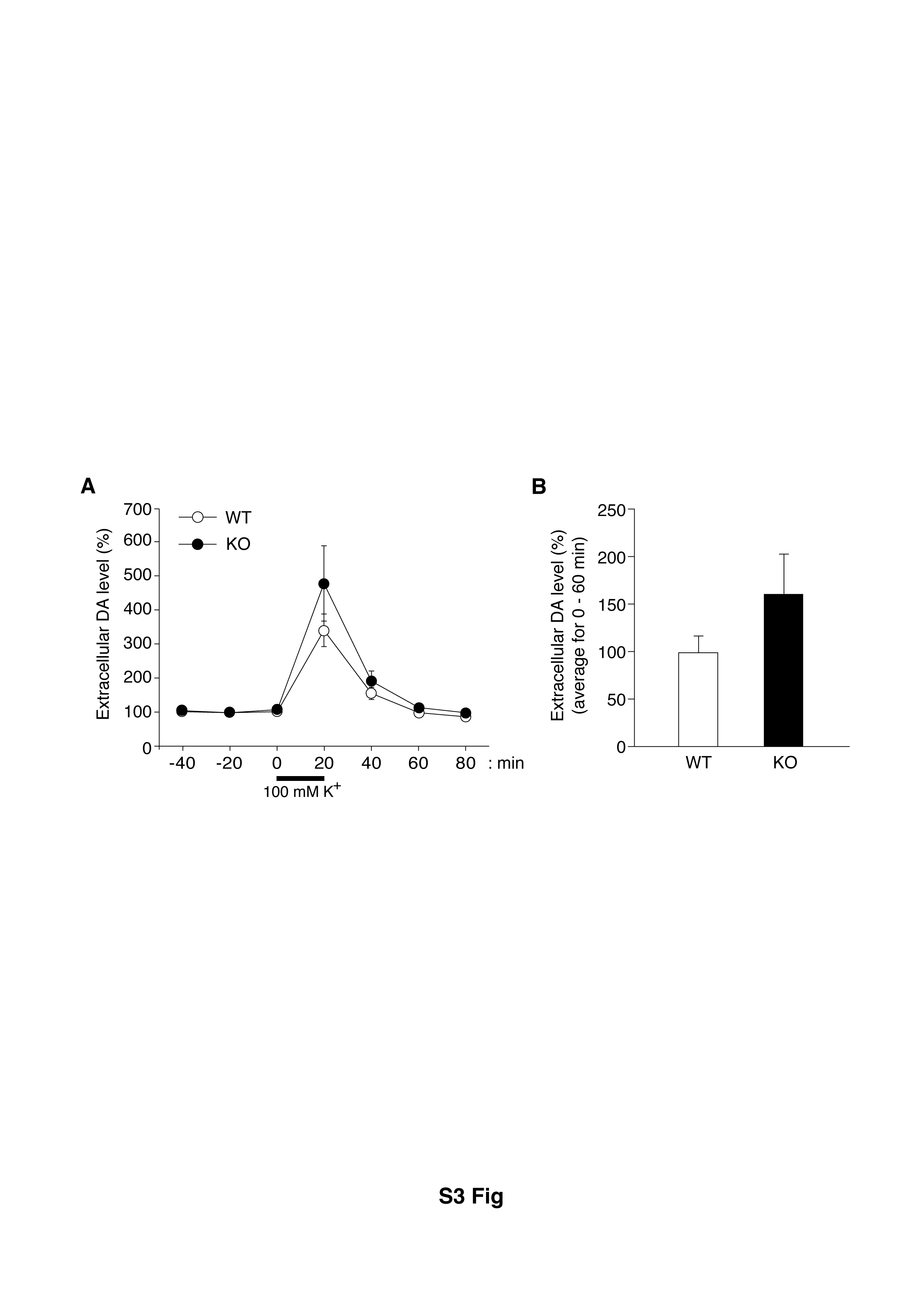

Supplement: S3 Fig — (A, B) Microdialysis measurements were performed as shown in Fig 6. Plots show the mean ± SE of wild-type and Ptprz-KO (n = 9–10 per group) (A). The horizontal bar indicates the application of 100 mM K+ solution (for 20 min) via the microdialysis probe. Dopamine levels were expressed as a percentage of averaged basal values (two points, -40 ~ -20 min and -20 ~ 0 min). Summary (B). The bar graph shows the mean with SE of extracellular DA levels for 60 min after the application of high K+ solution. There was no significant difference between the two genotypes (the Student’s t-test). (TIF) [file pone.0221205.s004.tif]

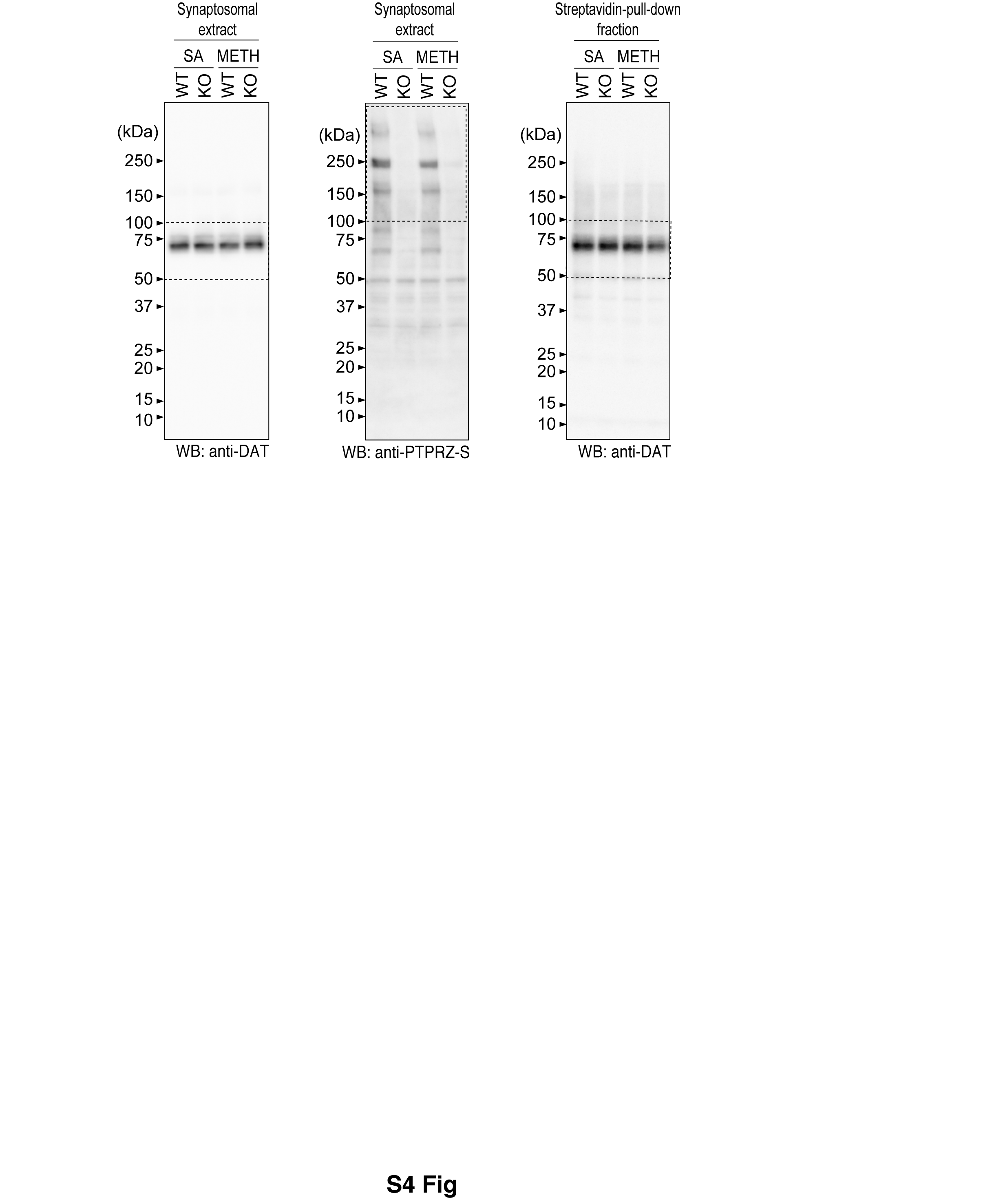

Supplement: S4 Fig — (TIF) [file pone.0221205.s005.tif]
