# Supplementary material for: Mice deficient in protein tyrosine phosphatase receptor type Z (PTPRZ) show reduced responsivity to methamphetamine despite an enhanced response to novelty
Source: PLoS One. 2019 Aug 20;14(8):e0221205. doi: 10.1371/journal.pone.0221205 (PMC6701799; doi:10.1371/journal.pone.0221205)
Supplement: S1 Text — (DOCX) [file pone.0221205.s006.docx]

**Supplementary Materials and Methods**

**Single cell RT-PCR**

We performed single-cell RT-PCR analyses as described previously [45] with some modifications. Briefly, the ventral tegmental area and substantia nigra tissues were dissected from coronal brain sections (400-µm-thick) and dissociated into single cells by pronase treatments and repeated pipetting. After allowing cells to settle on culture dishes, the contents of a single cell were aspirated into an electrode pipette, and immediately recovered into a tube containing 14 µl of ice-cold reaction buffer and 14 units RNasin (Promega corporation). First-strand cDNA synthesis was performed in a final volume of approximately 20 µl using the Superscript II reverse transcriptase kit (Life Technologies) with 5 ng of a random hexamer primer at 42°C for 50 min according to the manufacturer’s instructions, followed by an RNase (2 units) treatment at 37°C for 20 min.

The expression of tyrosine hydroxylase (Th), glutamate decarboxylase 1 (Gad1), glial fibrillary acidic protein (Gfap), and PTPRZ was then examined by two-stage PCR amplification using the primers listed in S2 Fig. First multiplex PCR was performed with a primer mixture for all four genes (listed in the upper portion of the table in S2 Fig) in a final volume of 20 µl containing 5 µl of single-cell cDNA, 20 pmol of each primer, 200 µM dNTPs, 2 µl of 10×PCR buffer, and 1 U of EX-Taq (Takara) under the following conditions: a 5-min denaturation at 94°C for 5 min, 25 cycles of 95°C for 30 sec, 62°C for 3 sec, and 72°C for 2 min, and a final extension at 72°C for 5 min. Nested-PCR was performed using each gene-specific primer set (listed in the lower portion of the table) in a final volume of 20 µl containing 0.1 µl of the first PCR product, 200 µM dNTPs, 2 µl of 10× PCR buffer, 0.5 U of EX-Taq, and 10 pmol of each second primer set under the following conditions: a 5-min denaturation at 94°C, 35 cycles of 95°C for 30 sec, 62°C for 3 sec, 72°C for 1 min, and a final extension at 72°C for 2 min. PCR products were separated on a 3% Nusive 3:1 agarose gel (FMC BioProducts) and visualized with ethidium bromide staining.

**Immunofluorescence analysis of primary cultured dopamine neurons**

Primary cultures of mouse mesencephalic neurons were performed as described previously [46]. Cells were fixed with 4% paraformaldehyde in PBS. After permeabilizing and blocking, cells were incubated with anti-DAT (1:1000) and anti-PTPTR-S (1:3000), followed by Alexa Fluor-conjugated secondary antibodies (Molecular Probes). Digital photomicrographs were taken with a confocal microscopy (LSM510, Carl Zeiss).

**Enzyme assays**

Tyrosine hydroxylase (TH) activity was assessed as described previously [47] with slight modifications. L-[3,5-^3^H]-tyrosine dried under a stream of nitrogen and reconstituted in a 500 µM solution of unlabeled tyrosine to 0.5 µCi/nmol. Brain tissues quickly separated on ice were homogenized with a 10-fold (*v*/*w*) solution of 50 mM Tris-HCl, pH 6.0 containing 0.2 % Triton X-100. A 10-µl aliquot was added to 100 µl of substrate solution (25 µM isotopic tyrosine in 50 mM MES-Tris buffer, pH 6.1 containing 5 mM DTT, 0.5 mM tetrahydro-L-biopterin (BH_4_), and 2000 units/ml catalase) and incubated at 37°C for 20 min. Blank values were obtained by omitting BH_4_. The reaction was terminated by adding 1 ml of 7.5% charcoal in 1 M HCl. Aliquots of the supernatant were mixed with 10 ml of Aquasol II for scintillation counting.

Choline acetyltransferase (ChAT) [48] and glutamate decarboxylase (GAD) [49] activities were assessed as described previously with modifications. Brain tissues were homogenized with a 10-fold (*v*/*w*) solution of 10 mM phosphatase buffer (PB), pH 7.0 and stored at -85°C until used. In the ChAT assay, 1-[^3^H]-acetyl-CoA was reconstituted with unlabeled acetyl-CoA to 6 to 7 µCi/nmol. Stored tissue homogenates were diluted 2-fold with 50 mM PB, pH 7.4 containing 20 mM EDTA, and this was followed by the preparation of a dilution series for appropriate quantitation with 50 mM PB, pH 7.4, containing 10 mM EDTA. A 5-µl aliquot was added to 30 µl of substrate solution (0.23 mM isotopic acetyl-CoA in 50 mM PB, pH 7.4, containing 233 mM NaCl, 5 mM EDTA, 2.33 mM choline chloride, 0.233 mM eserine sulfate, and 0.12% Triton X-100), and incubated at 37°C for 15 min. Blanks were incubated on ice. The reaction was terminated by adding 5 ml of 0.3 mM acetylcholine in 50 mM PB, pH 7.4, and the ^14^C-labeled product was separated from the precursor by extraction into 2 ml of 0.5% sodium tetraphenylborate in acetonitrile. Aliquots of acetonitrile extracts were mixed with 10 ml of toluene scintillation solution for counting.

In the GAD assay, L-[1-^14^C]-glutamate was reconstituted with unlabeled glutamate to 1 µCi/µmol, and GAD activity was measured as [^14^C]-CO_2_ production from L-[1-^14^C]-glutamic acid. Stored tissue homogenates were diluted 4-fold with 50 mM PB, pH 7.0, containing 10 mM 2-mercaptoethanol (2-ME) and 0.25% Triton X-100, and this was followed by the preparation of a dilution series for appropriate quantitation with 50 mM PB, pH 7.0, containing 7.5 mM 2-ME and 0.2% Triton X-100. A 5-µl aliquot was added to 15 µl of substrate solution (30 mM isotopic L-glutamic acid in 50 mM PB, pH 7.4, containing 0.3 mM pyridoxal), and incubated at 37°C for 20 min. Blanks were incubated on ice. The reaction was terminated by adding 50 µl of 1 M H_2_SO_4_. The release of [^14^C]-CO_2_ was measured by trapping CO_2_ with filter paper soaked in benzethonium hydroxide at 37°C for 60 min, and the radioactivity of each filter paper was measured by liquid scintillation counting.

**Supplementary References**

45. Surmeier DJ, Song WJ, Yan Z. Coordinated expression of dopamine receptors in neostriatal medium spiny neurons. J Neurosci. 1996;16(20):6579-91. Epub 1996/10/15. PubMed PMID: 8815934.

46. Yamamoto H, Kamegaya E, Hagino Y, Imai K, Fujikawa A, Tamura K, et al. Genetic deletion of vesicular monoamine transporter-2 (VMAT2) reduces dopamine transporter activity in mesencephalic neurons in primary culture. Neurochem Int. 2007;51(2-4):237-44. Epub 2007/08/01. doi: 10.1016/j.neuint.2007.06.022. PubMed PMID: 17664021.

47. Reinhard JF, Smith GK, Nichol CA. A rapid and sensitive assay for tyrosine-3-monooxygenase based upon the release of 3H2O and adsorption of [3H]-tyrosine by charcoal. Life sciences. 1986;39(23):2185-9.

48. Whittaker V, Barker L. The subcellular fractionation of brain tissue with special reference to the preparation of synaptosomes and their component organelles. Methods of neurochemistry. 1972;2:1-52.

49. Miller LP, Martin DL, Mazumder A, Walters JR. Studies on the regulation of GABA synthesis: substrate-promoted dissociation of pyridoxal-5'-phosphate from GAD. J Neurochem. 1978;30(2):361-9. Epub 1978/02/01. PubMed PMID: 24086.
